# Fluorescent water-soluble organic aerosols in the High Arctic atmosphere

**DOI:** 10.1038/srep09845

**Published:** 2015-04-28

**Authors:** Pingqing Fu, Kimitaka Kawamura, Jing Chen, Mingyue Qin, Lujie Ren, Yele Sun, Zifa Wang, Leonard A. Barrie, Eri Tachibana, Aijun Ding, Youhei Yamashita

**Affiliations:** 1 State Key Laboratory of Atmospheric Boundary Layer Physics and Atmospheric Chemistry, Institute of Atmospheric Physics, Chinese Academy of Sciences, Beijing 100029, China; 2 Institute of Low Temperature Science, Hokkaido University, Sapporo 060-0819, Japan; 3 SKLEG, Institute of Geochemistry, Chinese Academy of Sciences, Guiyang 550081, China; 4 Institute of Geographic Sciences and Source Research, Chinese Academy of Sciences, Beijing 100101, China; 5 Bolin Centre for Climate Research, Stockholm University, Stockholm 106 91, Sweden; 6 Institute for Climate and Global Change Research & School of Atmospheric Sciences, Nanjing University, Nanjing 210093, China; 7 Faculty of Environmental Earth Science, Hokkaido University, Sapporo 060-0810, Japan

## Abstract

Organic aerosols are ubiquitous in the earth’s atmosphere. They have been extensively studied in urban, rural and marine environments. However, little is known about the fluorescence properties of water-soluble organic carbon (WSOC) or their transport to and distribution in the polar regions. Here, we present evidence that fluorescent WSOC is a substantial component of High Arctic aerosols. The ratios of fluorescence intensity of protein-like peak to humic-like peak generally increased from dark winter to early summer, indicating an enhanced contribution of protein-like organics from the ocean to Arctic aerosols after the polar sunrise. Such a seasonal pattern is in agreement with an increase of stable carbon isotope ratios of total carbon (*δ*^13^C_TC_) from −26.8‰ to −22.5‰. Our results suggest that Arctic aerosols are derived from a combination of the long-range transport of terrestrial organics and local sea-to-air emission of marine organics, with an estimated contribution from the latter of 8.7–77% (mean 45%).

Atmospheric aerosols have been recognized to play an important role in regulating regional and global climate^1^. For example, the enrichment of aerosol particles with organic compounds can make the aerosol surfaces more hydrophilic or hydrophobic depending on the composition and mixing state, leading to alterations in the cloud condensation nuclei (CCN) activity of the particles. When deposited onto the earth’s surface, atmospheric aerosols can darken snow albedo, and modify both land and ocean biogeochemistry^2^. Thus, the long-range atmospheric transport of aerosol particulate matter from continents to open oceans^3^ or to the polar regions^4^,^5^ is an active area of research.

Water-soluble organic carbon (WSOC) is a major fraction of atmospheric aerosols. WSOC contains aromatic rings or aliphatic structures with carboxyl, hydroxyl, carbonyl or methoxy functional groups. The chemical nature of 10–20% (by mass) of WSOC has been resolved at a molecular level, and found to include mono- and di-carboxylic acids, sugar compounds, amino acids, and polar organic marker compounds (e.g. 2-methyltetrols and pinic acid) from the photooxidation of biogenic volatile organic compounds (BVOCs)^5^,^6^,^7^,^8^,^9^. The majority of WSOC is currently thought to be the water-soluble fraction of HUmic LIke Substances (HULIS), which are similar to terrestrial and aquatic humic and fulvic acids^10^,^11^.

Fluorescence techniques (e.g. synchronous scan and excitation-emission matrix spectroscopy) have been widely used to investigate the sources and optical properties of dissolved organic matter (DOM) or humic substances in aquatic environments^12^,^13^,^14^,^15^. Such techniques are widely applied to study the sources and chemical nature of chromophoric DOM (CDOM) in oceans^16^,^17^. For example, the use of excitation-emission matrix (EEM) spectroscopy permits discrimination of CDOM sources based on the relative abundances of different fluorophores, including humic-like, protein-like, and pigment-like fluorescence^12^,^17^. Many studies have suggested that the optical properties of chromophoric WSOC in the atmosphere may be similar to CDOM in aquatic environments^18^,^19^. Furthermore, fluorescence techniques have been used to study photo-degradation effects on CDOM in rainwater^20^ and WSOC in aerosol particles^11^,^19^,^21^,^22^,^23^. During the past few years, on-line fluorescence instruments, such as the ultraviolet aerodynamic particle sizer (UV-APS) and the wideband integrated bioaerosol sensor (WIBS) have been successfully used to measure emission spectra of single bioaerosol particles in ambient air^24^,^25^. Recent field and laboratory studies have also demonstrated that non-biological particles such as biogenic SOA derived from the photooxidation of isoprene and monoterpenes, can emit a strong fluorescence^26^,^27^.

The Arctic atmosphere was once considered as one of the most pristine environments on earth. However in the 1950s, two pilots flying over the North American Arctic noticed a widespread haze that was subsequently found to be observable every winter and early spring^4^. Since then, there has been much research into Arctic haze, which has now been elucidated as a mixture of sulfate, ammonium, nitrate, black carbon, organic carbon, that also contains relatively high levels of ozone precursors such as nitrogen oxides (particularly in the form of peroxyacetylnitrate, PAN) and volatile organic compounds (VOCs)^4^,^8^. During the polar sunrise season, the Arctic atmosphere is considered to serve as a unique photochemical reactor influenced by continent-derived particles and their precursors from the mid-latitudes in Eurasia or North America and marine-derived particles from the Arctic Ocean^4^. However, little is known about the changes in optical characteristics and sources of bulk organic aerosols before and after polar sunrise.

The objective of this study was to investigate the EEM properties of WSOC in Arctic aerosols collected at Alert, in the Canadian High Arctic (Figure 1a), in order to understand the influence of marine organics to organic aerosols in the Arctic atmosphere. The temporal trends of humic-like and protein-like fluorescence were compared with molecular marker compounds including methanesulfonic acid (MSA), a specific oxidation product of dimethyl sulfide (DMS) of marine origin^28^, and other polar organic species from biomass burning and biogenic VOC oxidation. In addition, stable carbon isotope ratios (*δ*^13^C) of High Arctic aerosols were determined to obtain new insights into the influence of sea-to-air emissions of marine organics to Arctic aerosol composition from dark winter to light spring.

## Results

### OC and WSOC concentrations

The concentrations of organic carbon (OC) and WSOC in the Alert aerosols ranged from 73.4–387ng m^−3^ (mean 253ng m^−3^) and 40.7–300ng m^−3^ (186ng m^−3^), respectively (Table 1). The aerosol mass concentrations ranged from 2540–9130ng m^−3^ (5250ng m^−3^). OC and WSOC made up only a small fraction of the aerosol mass (2.1–4.2% and 1.2–3.3%, respectively), suggesting that mineral sea salts dominate High Arctic aerosols. Their fractions were higher in February and late March, and lower in early summer. The decrease in the concentrations of OC and WSOC (Figure 2a–b) from February to early March is consistent with previous studies^29^,^30^.

### Stable carbon isotope ratios

Stable carbon isotopic composition (*δ*^13^C) has been successfully used to understand the contributions of marine and continental sources to aerosol carbon^29^,^31^,^32^. Based on *δ*^13^C measurements, *Chesselet* et al.^33^ reported that terrestrial organic matter is more important (>80%) in the remote marine aerosols than marine-derived organic matter. In the present study, the stable carbon isotope ratios, *δ*^13^C, in total carbon (TC) fall in a relatively narrow range of −22.5 to −26.8‰ (Table 1). The *δ*^13^C values before the polar sunrise ranged from −25.3 to −26.8‰, lower than those (−22.5‰ to −25.0‰) during light spring to early summer.

### Fluorescence properties of WSOC

**Figure 1:**
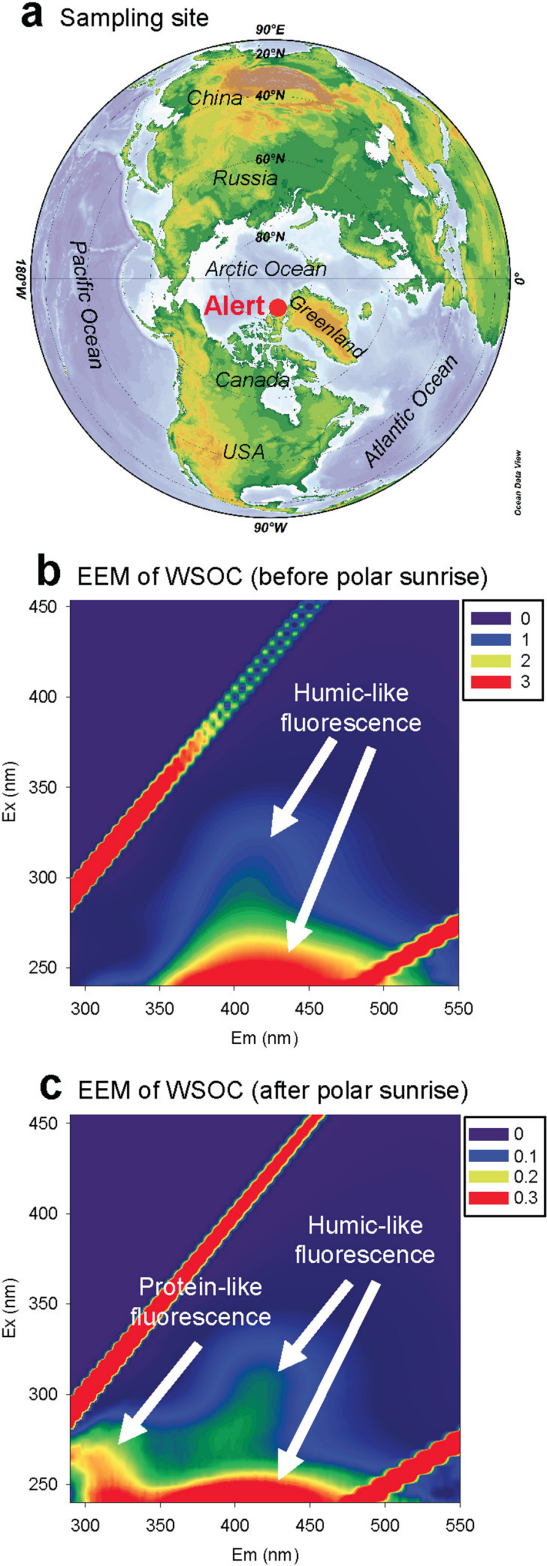
(a) A map showing the sampling site of Alert (82.5^°^N, 62.3^°^W) in the Canadian High Arctic; (b) and (c) are typical excitation-emission matrix (EEM) fluorescence spectra of water-soluble organic carbon (WSOC) in the aerosol samples collected before and after the polar sunrise, respectively. The map in the figure was drawn by Ocean Data View.

**Figure 2:**
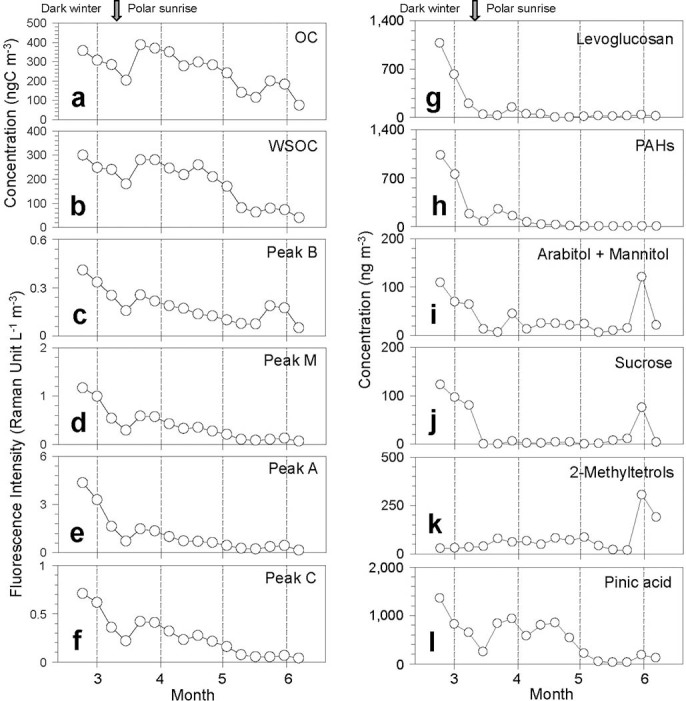
Temporal variations of (a) organic carbon (OC), (b) water-soluble organic carbon (WSOC), (c–f) fluorescence intensities, as well as (g–l) molecular marker compounds^5^,^8^ in the aerosol samples collected at Alert in the Canadian High Arctic. Levoglucosan is a tracer for biomass-burning emissions^3^; PAHs are anthropogenic tracers for fossil fuel combustion; arabitol and mannitol are tracers for atmospheric fungal spores^40^; sucrose is a tracer for primary bioaerosols such as pollen^41^; 2-methyltetrols and pinic acid are tracers for secondary organic aerosols from the photooxidation of isoprene and monoterpenes^7^,^9^, respectively.

Figure 1 presents typical EEM spectra of WSOC in High Arctic aerosols. Before the polar sunrise, strong humic-like fluorophores were observed at ex/em = 240–250/416–426nm and ex/em = 335–345/436–446nm, which are traditionally defined as Peak A and Peak C, respectively^12^. After the polar sunrise, an obvious peak was also found at ex/em = 270–280/310–320nm (Peak B), which is generally considered to correspond to protein-like fluorophores^12^. The fluorescence intensities of peaks A (terrestrial humic-like), C (terrestrial humic-like), M (marine humic-like), and B (protein-like) obtained from the excitation/emission pairs are summarized in Table 1. Their temporal variations (Figure 2c–f) are consistent with those of OC and WSOC (Figure 2a–b), decreasing from dark winter to early summer, with a pronounced dip in early March. Such decreases have also been observed in the temporal variations of biomass burning tracers (e.g. levoglucosan and vanillic acid) and fossil fuel combustion tracers (polycyclic aromatic hydrocarbons, PAHs)^5^, suggesting that the long-range atmospheric transport of continental aerosol from mid-latitudinal regions in the Northern Hemisphere is an important factor in controlling the abundances of fluorescent components in Arctic aerosols.

### Air masses transport characteristics

**Figure 3:**
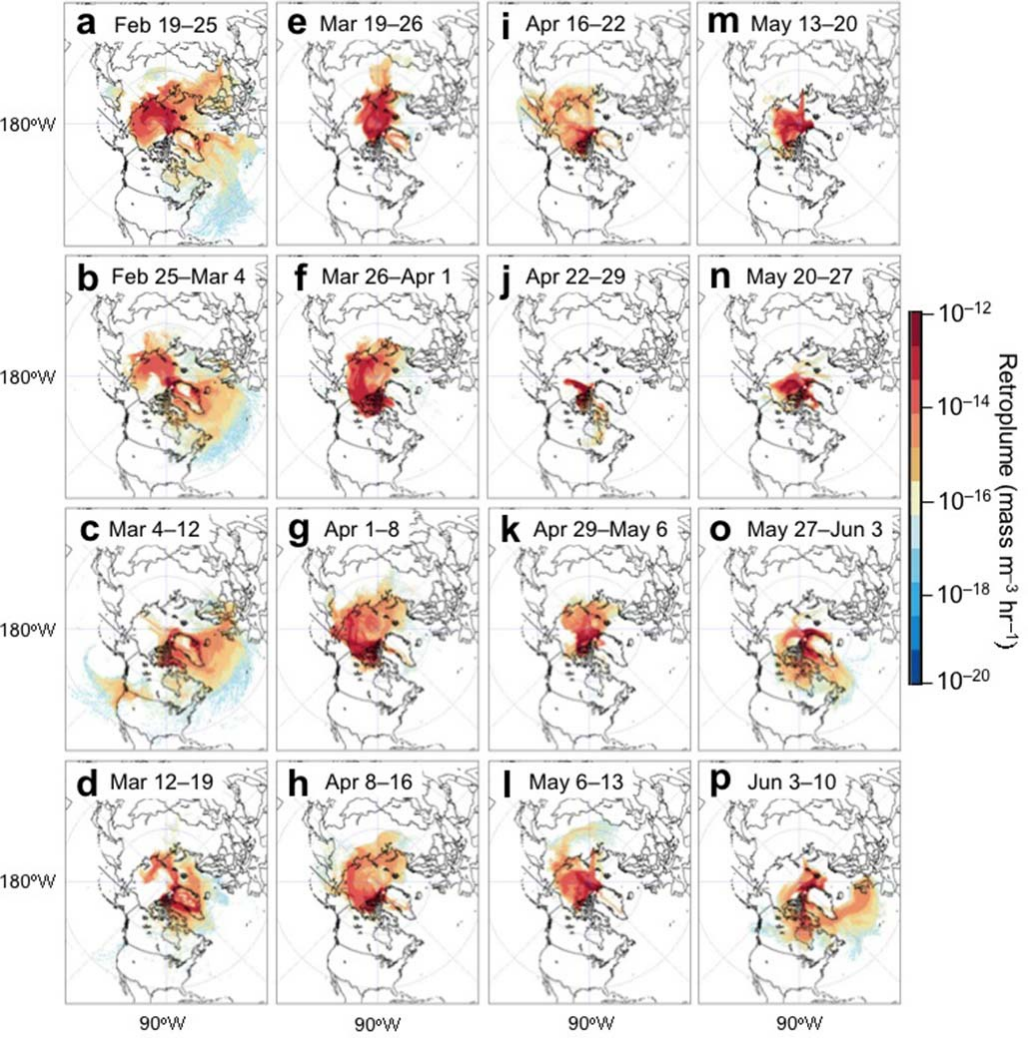
Averaged retroplumes (“footprint” residence time) showing the transport pathways of air masses observed at Alert (shown as a white circle on each map). The sampling period for each sample was about one week. The maps were drawn by the software of Igor Pro, http://www.wavemetrics.com/.

In order to understand the general transport characteristics of air masses recorded at the sampling site, we conducted a ten-day backward Lagrangian Particle Dispersion Modeling (LPDM) analysis using the Hybrid Single-Particle Lagrangian Integrated Trajectory (HYSPLIT) model^34^. This method has been shown to perform well in the simulation of long-living species such as CO, and has been widely used to understand the transport and origins of air pollutants^35^,^36^. Based on the backward particle release simulation for each hour during the sampling period, the average distribution of retroplumes for each sample collected at Alert in the High Arctic is shown in Figure 3. Before the polar sunrise, the air masses originated from the Arctic Ocean, Canadian High Arctic and Greenland. From late March to mid-May, the retroplumes show that the air masses were mainly delivered from the Arctic Ocean to the sampling site with a significant air mass transport from North America in late March. After late May, the air mass contribution from North America was enhanced.

## Discussion

The time series measurements of OC and WSOC (Figure 2a–b) showed a decrease from February toward mid-March. After the polar sunrise, their concentrations increased rapidly and remained high till mid-April. They then decreased in mid-May and slightly increased toward early June. Further, both the humic-like and protein-like fluorescence peaks (Figure 2c–f) showed a similar pattern, consistent with those observed for molecular marker compounds for primary organic aerosols (POA) that are directly emitted from sources such as plant material, soil dust, biomass/biofuel and fossil fuel combustion^5^. For example, during the same campaign, similar patterns were also observed for levoglucosan and polycyclic aromatic hydrocarbons (PAHs) (Figure 2g–h). Levoglucosan, a biomass-burning tracer that is produced in large quantities during the pyrolysis of cellulose^3^, was found in Arctic aerosols with large variations (3–1080pg m^−3^) but higher concentrations in dark winter. It is not surprising given that biomass-burning emissions are one of the most significant sources for atmospheric HULIS^10^,^37^. PAHs are mainly representative of anthropogenic aerosols, which are produced from fossil fuel combustion and biomass burning.

The winter maxima of OC, WSOC, and organic species can be explained by the concept of the Arctic acting as a cold sink during winter to receive long-range transported aerosols and their precursors transported from their emission regions in the mid-latitudes in Eurasia and North America. Under stagnant conditions with the lack of solar radiation in winter, aerosol removal rates are minimized^38^. After the polar sunrise, the increased concentrations of OC and WSOC are a result of the enhanced sea-to-air emissions of marine organic matter, and the production of secondary organic aerosols (SOA) from the photooxidation of anthropogenic and biogenic VOCs.

Fluorescent aerosol particles may also contain primary biological aerosol particles, which are ubiquitous and comprise a variety of particle types such as bacteria, algae, pollen, fungal spores, plant debris and biopolymers (protein, DNA, chitin, cellulose and other polysaccharides)^24^,^25^,^39^. In the present study, the fungal spore tracers, arabitol and mannitol^40^, showed a similar pattern to levoglucosan and PAHs from February to May. However, a significant increase was then observed at the end of May when the air masses mainly originated in North America (Figure 3m–p). Sucrose, a tracer for airborne pollen^41^, also peaked in late spring. Such an enhancement of protein-like fluorescence was also observed during late May to early June (Figure 2c). Thus, the similarity in temporal variations between fluorescence peaks and POA tracers indicates that biomass burning, fossil fuel combustion and primary biological aerosols are the main sources of fluorescent organics in the Arctic regions, and that they may be co-transported long distances from the mid-latitudinal regions. For example, *Rousseau et al.*^42^ reported the long-distance transport of pollen grains from boreal forests in northeastern North America to Greenland in spring.

In the Earth’s atmosphere, SOA formed by the photooxidation of BVOCs is believed to be more abundant than directly emitted POA. Recent field and laboratory studies have demonstrated that non-biological particles such as biogenic SOA from the photooxidation of isoprene and monoterpenes can also emit a strong fluorescence^26^,^27^. In a previous study, *Fu et al.*^8^ reported the temporal variations of biogenic SOA tracers, which show that monoterpene SOA tracers such as pinic acid (Figure 2l) exhibited temporal patterns similar to those of OC and WSOC. β-caryophyllinic acid, a specific tracer for the photooxidation of β-caryophyllene^43^ (a sesquiterpene known to have a high aerosol yield), also followed such a trend^8^. Positive correlations between fluorescence peaks and monoterpene and β-caryophyllene oxidation products indicate an intrinsic relationship between biogenic SOA and these fluorescent components.

The temporal variations of 2-methyltetrols (Figure 2k), the isoprene oxidation products, were substantially different from those of OC, WSOC, monoterpene- and sesquiterpene-SOA tracers, and fluorescent peak intensities. The general increase of isoprene oxidation products during February-May is most likely caused by local and/or regional emissions of isoprene from the open ocean followed by photooxidation. Isoprene emitted from marine algae could be a significant precursor of marine SOA^44^. The biologically active waters of the adjacent seas and open oceans in the Arctic have been proposed as a source of organic aerosols in summer^45^. The sharp increase of 2-methyltetrols together with molecular markers of fungal spores and pollen in late May (Figure 2i–k) may be caused by the shift in source regions of Arctic aerosols. The LPDM simulations have shown that in mid-May the air masses mainly originated from the Arctic Ocean and north Greenland, whereas in late May they mostly came from North America across the Baffin Bay (Figure 3m–p).

**Figure 4:**
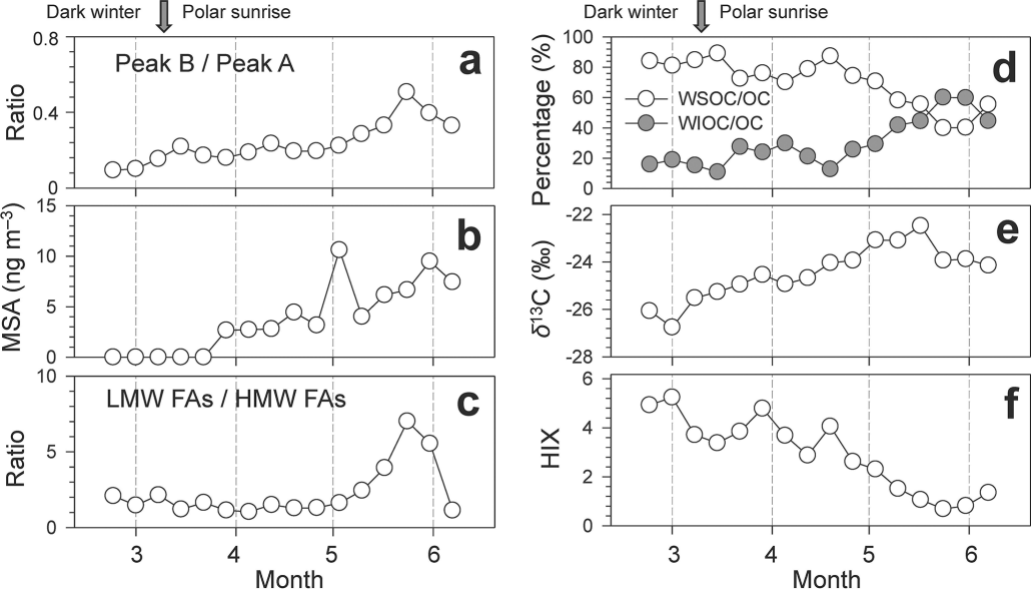
Temporal variations of some major parameters measured in the aerosol samples collected at Alert in the Canadian High Arctic. Data of methanesulfonic acid (MSA) are from *Kawamura et al.*^6^.

Furthermore, an increase in the relative enhancement of protein-like fluorescence (peak B) to humic-like fluorescence (peak A) in the WSOC fraction (Figure 4a) from dark winter to light spring may indicate the enhancement of sea-to-air emission of marine biota-derived organics in spring, although such an increase of peak B / peak A may also partly result from the different photodegradation rates of humic-like substances and protein-like substances (humic type organics are considered to be more photoreactive than phytoplankton derived organics^46^). However, the interpretation of the sea-to-air emission of marine organics as the cause of the enhancement is supported by an increase in methanesulfonic acid (MSA) (Figure 4b) in the same aerosol samples^6^. MSA is a photooxidation product of dimethylsulfide of marine algal origin^28^. In fact, marine biological activities can emit a large amount of primary organic aerosols, together with biogenic VOCs, into the marine atmosphere. The enhanced contribution of marine sources is also supported by the concentration ratios of lower molecular weight (LMW) to higher molecular weight (HMW) fatty acids. LMW fatty acids (i.e. those with carbon numbers <C_20:0_) are a major fraction of marine aerosols , while HMW fatty acids (>C_20:0_) mainly originate from terrestrial higher plants. The concentration ratios of LMW to HMW fatty acids in High Arctic aerosols showed an increased trend from February to May (Figure 4c), ranging from 1.0 to 7.0 with an average of 2.3 (Table 1); the ratio decreased under the influence of the air masses transported from North America in late May (Figure 3).

It has been recognized that in the marine atmosphere, organic enrichment is strongly associated with decreasing size of particles^47^. For example, size distributions of organic carbon in marine aerosols have shown that both WSOC and WIOC (water-insoluble organic carbon) substantially existed in the fine submicron fraction^47^. In the High Arctic region, the photochemically produced components of SOA should be enhanced after the polar sunrise. This is especially true for the photooxidation products of oxalic acid and other dicarboxylic acids in Arctic aerosols with an enrichment in the fine fraction^6^. SOA is generally considered to be a major fraction of WSOC. Thus, the contribution of WSOC to total OC should be enhanced in spring-summer in the High Arctic under strong sunlight irradiation. However, a decrease in the contribution of WSOC to OC was observed between February to June (Figure 4d), suggesting that the contribution of WIOC (WIOC = OC – WSOC) increased from dark winter to light spring, which indicates that primary marine organic aerosols generated from sea spray constitute a large fraction of WIOC.

**Figure 5:**
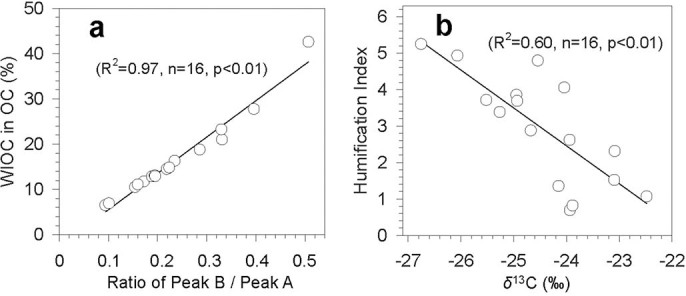
Linear correlations between (a) the ratio of peak B to peak A, and the percentage of water-insoluble organic carbon (WIOC) in OC, and (b) stable carbon isotope ratios (*δ*^13^C), and the humification index.

In fact, ocean surface waters are enriched with small particulate materials including phytoplankton, algae, bacteria, viruses, fragments of larger organisms and organic detritus^48^, which are known to be the main sources of marine microgels. Phytoplankton exudates include exopolymer gels consisting of polysaccharides that bind together with LMW organics such as amino acids and peptides. Exopolymer gels are insoluble, thermally stable, highly surface active and hydrated^48^. Thus, the pool of organic matter in the marine surface layer contains not only dissolved organic matter but also water-insoluble organics^48^ that are emitted into the marine boundary layer (MBL) as sea spray via bubble bursting and breaking waves. For example, *Hawkins and Russell*^49^ reported that freshly emitted organics from the ocean likely have low water solubility. *Facchini et al*.^50^ also reported that primary organic aerosols emitted with sea sprays in a more biologically active ocean are comprised of water-insoluble organics (up to 77 ± 5%), which contain phytoplankton-derived detritus. The strong positive correlation between WIOC contribution to OC and the ratio of protein-like fluorescence to humic-like fluorescence (peak B / peak A) (Figure 5a) suggests an obvious increase of WIOC materials in the High Arctic atmosphere due to the sea-to-air emission of marine organics. In a previous study, marine microgels were abundantly identified in aerosols, fog and cloud waters in the MBL over the Arctic Ocean in summer^51^.

Based on ionic composition and stable carbon isotope ratios (*δ*^13^C) of Arctic aerosols collected during the ALERT2000 campaign, *Narukawa et al.*^29^ postulated an increase in biogenic emissions from the surrounding open ocean in late spring to early summer in the Arctic. The enhanced sea-to-air emission of marine organics from February to June was also supported by the temporal trend of *δ*^13^C values in High Arctic aerosols (Figure 4e), ranging from −26.8‰ to −22.5‰ (mean −24.5‰) (Table 1). The isotopic compositions of marine and continental carbonaceous aerosols have been well studied^31^,^32^,^33^. In general, *δ*^13^C values of TC for continental aerosols vary significantly between pollution sources. For example, *δ*^13^C values of primary aerosol particles emitted from fossil fuel combustion or biomass burning of C_3_ plants range from −24 to −37‰^29^,^31^,^32^,^33^,^52^,^53^; those of biogenic SOA from β-pinene ozonolysis have been reported to be −29.6 ± 0.2‰^54^; and of marine-derived particles are −20 to −22‰^33^. In order to estimate the relative contribution of marine and continental influences on the Arctic aerosols, the *δ*^13^C values in Figure 4e were applied to the following isotopic mass balance equations: 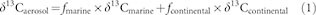

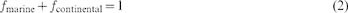
 where *f*_marine_ and *f*_continental_ are the fractions of marine and continental carbon, and *δ*^13^C_marine_ and *δ*^13^C_continental_ are the reported isotopic values for marine and continental carbon. Based on the previous studies^29^,^52^, the representative *δ*^13^C values of continental and marine aerosols were taken to be −27.3‰ and −21.0‰, respectively.

**Figure 6:**
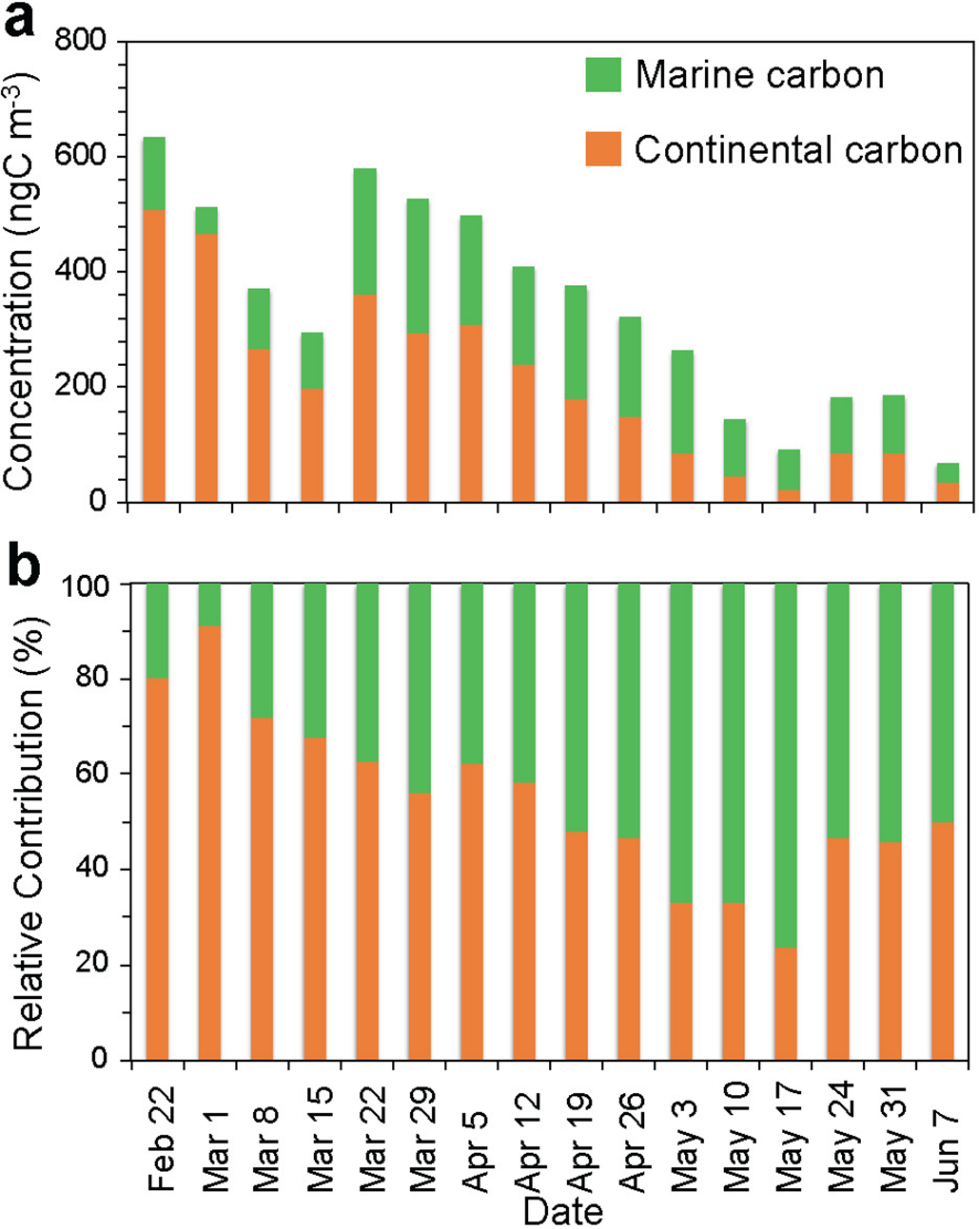
(a) Concentrations of marine and continental carbon estimated using stable carbon isotope ratios of total carbon (δ^13^C_TC_), and (b) the relative contributions of marine and continental carbon to High Arctic aerosols.

The contributions of marine carbon to High Arctic aerosols are estimated to be 8.7–77% with an average of 45%, which are consistent with those (0–67%, mean 45%) during the Alert2000 campaign^29^. Our estimates of marine carbon contribution for light spring aerosols (37–77%, 53%) are higher than that (38%) reported at Bermuda^52^, where the marine aerosols are more affected by anthropogenic sources than the Arctic. In a coastal urban site in Chennai, south India, the estimated contributions of marine carbon to urban aerosols were 1–37% (mean 19%)^55^, which are similar to those (8.7–32%, 22%) in the High Arctic during dark winter when the marine carbon can be expected to be transported from lower latitudinal marine regions. Figure 6a shows the concentrations of aerosol carbon from marine and continental sources calculated using the two end-member model (Eqns 1 and 2). The estimated marine carbon concentration ranged from 34.5 to 231ngC m^−3^ (mean 133ngC m^−3^) and continental carbon concentration from 21.7 to 510ngC m^−3 ^(210ngC m^−3^). The relative contribution of marine carbon clearly increased from winter to early summer (Figure 6b), indicating an enhanced sea-to-air emission of marine organics in the Arctic.

Ratios of fluorescence intensity from specific spectral regions of an EEM are often used as indicators for the relative contribution of organic matter derived from terrestrial or microbial sources in natural waters^13^,^14^,^56^,^57^. The humification index (HIX) was introduced by *Zsolnay et al.*^56^ to estimate the degree of maturation of DOM in soil. During the humification process, the aromaticity of organic matter increases and its microbial availability decreases. Thus, high HIX values (>10) correspond to strongly humified or aromatic organics, principally of terrestrial origin, while low values (<4) are indicative of autochthonous or microbial origin^14^,^15^,^57^. From February to June, a decrease of HIX for WSOC was observed, ranging from 5.2 to 0.7 (Figure 4f). In addition, a strong linear correlation (R^2^ = 0.60) between HIX and stable carbon isotope ratios was found in the Arctic samples (Figure 5b). These results again demonstrate a decreased contribution of terrestrial organics and an increased contribution of freshly emitted marine organics to Arctic aerosols from dark winter to light spring.

*McKnight et al.*^13^ proposed the fluorescence index (FI) as a proxy for the relative amount of DOM derived from terrestrial and microbial/algal sources in surface waters. FI values of 1.4 or less correspond to terrestrially derived organics and higher aromaticity, while values of 1.9 or higher correspond to microbial sources and lower aromatic carbon content^13^. Another index, the biological index (BIX)^57^, also allows an estimation of the contribution of autochthonous biological activity. An increase in BIX is related to an increase in the contribution of microbially derived organics. For example, high values (>1) have been shown to correspond to a predominantly biological or microbial origin of DOM and to the presence of OM freshly released into water, whereas values of <0.6 contain little biological material^57^.

**Figure 7:**
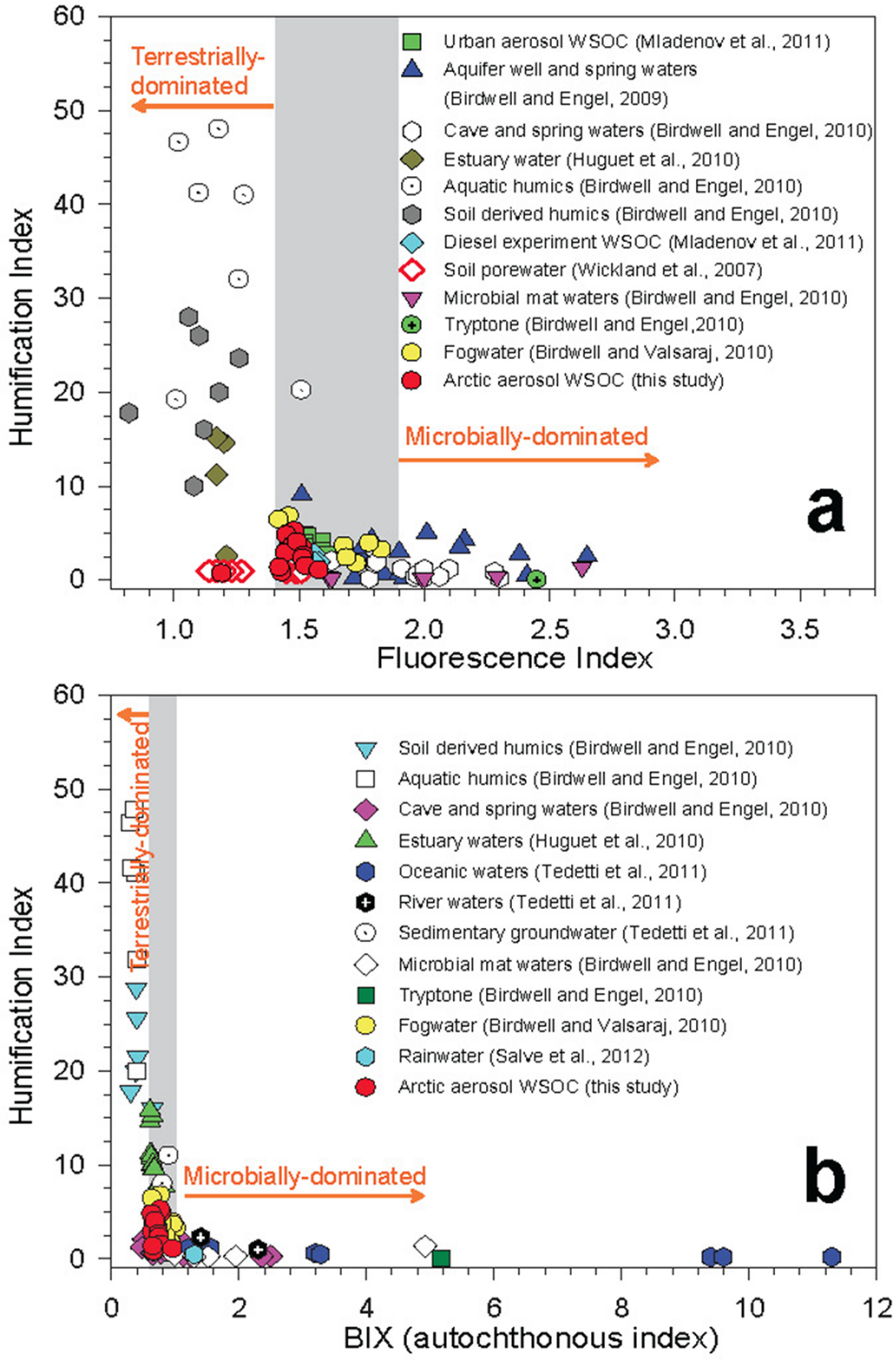
Comparison plots of humification index (HIX) versus (a) fluorescence index (FI) and (b) biological index (BIX) for the Arctic aerosol WSOC (red circles), together with different types of dissolved organic matter (DOM) that are adapted from *Birdwell and Engel*^14^,^59^, *Birdwell and Valsaraj*^60^, *Huguet et al*.^61^, *Mladenov et al*.^53^, *Salve et al*.^62^, *Tedetti et al.*^15^, and *Wickland et al*.^63^. The shaded regions represent mixed DOM signatures.

The FI and BIX values obtained from the Arctic aerosols are summarized in Table 1. The FI values ranged from 1.19 to 1.58 with an average of 1.47, indicating that the fluorophores in High Arctic aerosols are representative of both terrestrially and microbially derived organic matter. The BIX values (0.6–0.96, mean 0.72) are within the extreme values for the predominance of humic- or protein-like fluorophores. Interestingly, *Lee et al.*^26^ reported that the BIX values of SOA samples were on average 0.6. In High Arctic aerosols, the presence of marine emitted organics enhances the values of BIX, while biogenic SOA formed from the photooxidation of biogenic VOCs after the polar sunrise may contribute to the lower BIX values. Figure 7 plots the HIX data from this study as a function of FI and BIX, together with literature data for soil and aquatic humic substances, DOM in river and oceanic waters, as well as rain and fogwater samples^14^,^15^. The fluorescence indices of the High Arctic aerosol samples fall between those of terrestrially and microbially derived organics, which again suggests that High Arctic aerosols are a mixture of organics from continental aerosols brought to the region through long-range transport and marine organics emitted locally from the ocean surface layer.

## Methods

### Sample collection

Total suspended particulate matter (TSP) was collected weekly by filtering air through precombusted (450°C, 3h) quartz fiber filters using a high-volume sampler in the Canadian High Arctic at Station Alert (82.5°N, 62.3°W) from February 19 to June 10, 1991. The surface air temperature (mean) ranged from −33.5°C in February to −1.9°C in June during the sampling period. Each filter was stored in a precombusted glass bottle with a Teflon-lined screw cap in darkness at −20°C until analysis. Based on the reanalysis for dicarboxylic acids in 2010 of one aerosol filter sample (QFF 298) collected in mid-May during this campaign, and a comparison with the results obtained in 1994 for the same sample, *Kawamura et al.*^6^ found that the concentrations of major organic acids are equivalent within an analytical error of 15%. This suggests no serious degradation of organics during the sample storage. The experiments, including fluorescence and UV-Vis absorbance analysis, were finished in 2010.

### Air mass transport features

We conducted backward particle release simulations using the Lagrangian dispersion model Hybrid Single-Particle Lagrangian Integrated Trajectory (HYSPLIT), which was developed in the Air Resource Laboratory of the National Oceanic and Atmospheric Administration (NOAA), USA^34^. Here, we used ten-day backward Lagrangian Particle Dispersion Modeling (LPDM), as developed by *Ding et al.*^36^. In brief, for each hour during the sampling period, the model was run backward for ten days with 3000 particles released 100 m above ground level over the sampling site. The hourly position of each particle was calculated using a 3-D particle method. The spatiotemporal distributions of these particles were used to identify “footprint” retroplume and to calculate the potential source contributions^36^.

### WSOC extraction and analysis

An aliquot (ca. 10cm^2^) of filter samples were extracted by 20mL of organic-free Milli-Q water under ultrasonication for 20 min. The water extracts were then passed through a syringe filter (Millex-GV, 0.22μm, Millipore) and analyzed for water-soluble organic carbon (WSOC) using a TOC analyzer (Model TOC-Vcsh, Shimadzu, Japan).

### Fluorescence characterization

An aliquot (ca. 7cm^2^) of the filter samples were extracted by organic-free Milli-Q water under ultrasonication for 15 min. The water extracts were then passed through a syringe filter (Millex-GV, 0.22μm, Millipore). Excitation-emission matrix (EEM) fluorescence was measured using a fluorometer (Fluoromax-4, Horiba). All fluorescence spectra were acquired in the S/R mode with instrumental bias correction. Inner filter correction^13^ was carried out using absorbance spectrum measured with a spectrophotometer (Hitachi U-2000, Japan). After this procedure, the EEM of Milli-Q water was subtracted from the sample EEM. Finally, each EEM was calibrated to the water Raman signal^58^, and the fluorescence is in Raman units (RU, nm^−1^). Based on the ranges of *Coble*^12^, the fluorescence intensities of peaks A (terrestrial humic-like), C (terrestrial humic-like), M (marine humic-like), and B (protein-like) obtained from excitation/emission pairs are given in Table 1. Here the fluorescence intensity was further corrected to the amount of Milli-Q water used for the extraction and the air volume of each filter sample. Thus, the fluorescence is reported with a unit of RU L^−1^ m^−3^. In addition, the humification index (HIX, the ratio of H/L, where H is the fluorescence intensity recorded at Ex = 255nm for Em spectrum integrated from 434 to 480nm, and L is the fluorescence intensity recorded at Ex = 255nm for Em spectrum integrated from 300 to 434nm), index of recent autochthonous contribution (BIX, ratio of emission intensities at Em = 380nm and 430nm with excitation at Ex = 310nm) and fluorescence index (FI) (ratio of Ex/Em = 370/470nm to Ex/Em = 370/520nm) were calculated from each EEM^14^,^57^. The analytical uncertainty in the calculation of HIX, FI and BIX did not exceed 2%.

### Stable carbon isotope ratio measurement

Small punches (3.14cm^2^) of each quartz fiber filter sample were analyzed for total aerosol carbon content using an elemental analyzer (EA) (Carlo Erba, NA-1500). The filter punch was rounded using a pair of flat tipped tweezers, placed into a tin cup, and then caked into a ball. Before use, the tin cup was washed with acetone under ultrasonication to remove organic and other contaminants. Carbon was converted to CO_2_ in the combustion furnace (1020°C). Stable carbon isotopic analyses were conducted using the same EA interfaced (ThermoQuest, ConFlo II) to isotope ratio mass spectrometer (IRMS) (ThermoQuest, Delta Plus). The isotopic compositions (δ^13^C) were determined using the standard isotopic conversion: 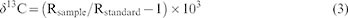
where R is the ratio of 13C/^12^C. Vienna Pee Dee Belemnite (^13^C) was used as the isotope standard. Samples were analyzed in duplicate and averaged concentrations and isotopic ratios are reported here after the blank correction. Reproducibility of TC measurement was within 2%. Analytical error in the carbon isotope ratios was within 0.2‰.

### Organic molecular marker measurement

The dataset of molecular marker compounds and detailed measurement methods have been reported elsewhere^5^,^8^. Briefly, a filter aliquot (ca. 20cm^2^) was cut into pieces and extracted three times with dichloromethane/methanol (2:1; v/v) under ultrasonication for 10 min. The solvent extracts were concentrated by a rotary evaporator, and then blown down to dryness with pure nitrogen gas. The extracts were then reacted with 50μL of N,O-bis-(trimethylsilyl)trifluoroacetamide (BSTFA) with 1% trimethylsilyl chloride and 10μL of pyridine at 70°C for 3 h. After reaction, the derivatives were diluted using 140μL of *n*-hexane with 1.43ng μL^−1^ of the internal standard (C_13_ *n*-alkane) prior to gas chromatography/mass spectrometry (GC/MS) injection. GC/MS analyses were performed on a Hewlett-Packard model 6890 GC coupled to Hewlett-Packard model 5973 mass-selective detector (MSD). The GC was equipped with a split/splitless injector and a DB-5MS fused silica capillary column (30m × 0.25mm i.d., 0.25μm film thickness). The GC oven temperature was programmed to increase from 50°C (2 min) to 120°C at 15°C min^−1^ and then to 300°C at 5°C min^−1^ with a final isotherm hold at 300°C for 16 min. Helium was used as the carrier gas at a flow rate of 1.0mL min^−1^. The sample was injected on a splitless mode with the injector temperature at 280°C. The mass spectrometer was operated on the electron impact (EI) mode at 70eV and scanned from 50 to 650 Da. Data were processed with the Chemstation software. Individual compounds were identified by comparison of mass spectra with those of authenticated standards or literature data. Field and laboratory blank filters were treated as the real samples for quality assurance. Target compounds reported here were not detected in the blanks. Relative standard deviations of the concentrations based on duplicate analysis were <10%.

## Figures and Tables

**Table 1:** Ambient temperatures, concentrations of major components, stable C and N isotope ratios, and fluorescence spectral parameters of Arctic aerosols.

Components	Min	Max	Mean	Median
Ambient temperature, °C	−35.0	−2.0	−19.6	−24.0
Aerosol mass, ng m^−3^	2540	9130	5250	5520
*δ*^13^C of TC (*δ*^13^C_TC_), ‰	−26.8	−22.5	−24.5	−24.3
OC, ngC m^−3^	73.4	387	253	280
WSOC, ngC m^−3^	40.7	300	186	214
WIOC, ngC m^−3^	22.0	120	68.0	58.6
WSOC in OC, %	39.9	89.1	69.9	73.4
WIOC in OC, %	10.9	60.1	30.1	26.6
LMW fatty acids (C_8_–C_19_), pg m^−3^	450	1680	929	807
HMW fatty acids (C_20_–C_30_), pg m^−3^	176	937	511	503
Ratios of LMW to HMW fatty acids	1.0	7.0	2.3	1.6
Marine Carbon, ngC m^−3^	34.5	231	133	115
Continental Carbon, ngC m^−3^	21.7	510	209	190
Fluorescence intensity, RU L^−1^ m^−3^				
peak B (protein-like, ex/em = 270–280/310–320nm)	0.010	0.084	0.035	0.034
peak M (marine humic-like, ex/em = 305–315/410–420nm)	0.013	0.239	0.074	0.059
peak A (terrestrial humic-like, ex/em = 240–250/416–426nm)	0.030	0.889	0.211	0.132
peak C (terrestrial humic-like, ex/em = 335–345/436–446nm)	0.008	0.145	0.050	0.043
HIX (ex = 255 nm, 436–480/300–344 nm)	0.69	5.24	2.93	3.12
FI (ex = 370, 470/520 nm)	1.19	1.58	1.47	1.48
BIX (ex = 310 nm, 380/430 nm)	0.63	0.96	0.72	0.70
